# Infectious Bursal Disease Virus in Algeria: Persistent Circulation of Very Virulent Strains in Spite of Control Efforts

**DOI:** 10.3390/ani14233543

**Published:** 2024-12-08

**Authors:** Chafik Redha Messaï, Nadia Safia Chenouf, Oussama Khalouta, Abdelhafid Chorfa, Omar Salhi, Claudia Maria Tucciarone, Francesca Poletto, Giovanni Franzo, Chahrazed Aberkane, Mattia Cecchinato, Matteo Legnardi

**Affiliations:** 1 Laboratory of Research Health and Animal Production, High National Veterinary School, Issad Abbes Street, Oued Smar, Algiers 16000, Algeria; 2 Faculty of Natural and Life Sciences, Earth and Universe Science, University Mohamed El Bachir El Ibrahimi of Bordj Bou Arreridj, El Anasser, Bordj Bou Arreridj 34000, Algeria; 3 Laboratory for Exploration and Valorization of Steppe Ecosystems (EVES), Department of Biology, Faculty of Natural Sciences and Life, University of Djelfa, Moudjbara Road BP 3117, Djelfa 17000, Algeria; 4 Laboratory of Life Sciences and Techniques, Institute of Agricultural and Veterinary Sciences, University Mohamed Cherif Messaadia, Souk Ahras 41000, Algeria; o.khalouta@univ-soukahras.dz; 5 Veterinary Office, Specialized in Avian Diseases and Analyses, El Eulma, Setif 19000, Algeria; chorfa.abdelhafid@gmail.com; 6 Biotechnology Laboratory of Animal Reproduction, Institute of Veterinary Sciences, Blida 09000, Algeria; dr.salhi-omar@hotmail.com; 7 Department of Animal Medicine, Production and Health, University of Padova, 35020 Legnaro, Italy; claudiamaria.tucciarone@unipd.it (C.M.T.); francesca.poletto.1@phd.unipd.it (F.P.); giovanni.franzo@unipd.it (G.F.); matteo.legnardi@unipd.it (M.L.); 8 DEDSPAZA Laboratory, Department of Agricultural Sciences, Mohamed-Khider University, Biskra 07000, Algeria; chahrazed2011@hotmail.com; 9 Department of Comparative Biomedicine and Food Science (BCA), University of Padova, 35020 Legnaro, Italy

**Keywords:** IBDV, molecular epidemiology, broiler, vaccination, Algeria

## Abstract

Infectious bursal disease (IBD) is among the most impactful immunosuppressive diseases of poultry. Its agent, infectious bursal disease virus (IBDV), is prone to both mutation and reassortment, resulting in a remarkable variability. Traditionally, IBDV characterization relies on antigenicity and pathogenicity assessment, but multiple phylogenetic classifications have been recently proposed, whose implementation in molecular surveys helps generating informative and standardized epidemiological data. In the present study, the Algerian IBDV scenario was assessed based on the novel classification guidelines by sequencing portions of both genome segments. Seventy pools of bursal samples were collected in 2022–2023 in 11 districts of Northern Algeria, mostly from broiler flocks. Out of 55 (78.6%) positive flocks, 40 (57.1%) were infected by field strains, which were characterized as very virulent strains (genotype A3B2) and phylogenetically related to previously reported Algerian strains. Significant differences in the percentage of field infections were observed between vaccinated (25/52, 46.2%) and unvaccinated (14/17, 82.3%) groups, and also between birds immunized with live intermediate (13/20, 65.0%) and intermediate plus (10/28, 35.7%) vaccines. Nonetheless, the number of field strain detections suggests a high infectious pressure and the inadequacy of current vaccination efforts, demanding a reevaluation of control measures coupled with attentive monitoring activities.

## Simple Summary

Infectious bursal disease (IBD) is one of the most relevant viral diseases of poultry, whose impact is associated both to clinical outbreaks and immunosuppression. It is caused by infectious bursal disease virus (IBDV), which features a significant genetic variability. To classify the many existing IBDV types, several phylogenetic schemes have been proposed in recent years, prompting the execution of molecular surveys to gather informative and standardized data. This study provides an epidemiological update of the Algerian situation based on molecular analyses of 70 bursal samples collected in 2022–2023. Among the 55 positive flocks (78.6%), 40 (57.1%) were infected with field strains, which were all characterized as very virulent IBDVs and had features consistent with other Algerian strains. The percentage of field strains in vaccinated flocks was significantly lower than unvaccinated ones, and differences in efficacy were also noted between different vaccine types. Despite this partial success in preventing field strain infections, current vaccination approaches do not seem sufficient to reduce the high IBDV infectious pressure in Algeria, demanding further efforts to ensure proper control and monitoring of the disease.

## 1. Introduction

Infectious bursal disease (IBD), also known as Gumboro disease, is a viral disease of chickens with a worldwide distribution. Its high economic burden is associated both to overt clinical outbreaks, usually observed in chickens from 3 to 6 weeks of age, and subclinical forms, mostly occurring in birds infected at an early age. Whilst inconspicuous, the latter still result in severe and prolonged immunosuppression, leading to increased secondary infections, worse performance, and vaccine failures [1]. In fact, IBD affects lymphoid tissues and has the bursa of Fabricius as its primary target, causing apoptosis and necrosis of immature B cells [2].

The causative agent of IBD is infectious bursal disease virus (IBDV), a non-enveloped, polyploid virus. IBDV is ascribed to the genus *Avibirnavirus* of the *Birnaviridae* family [3]. Its double-stranded RNA genome codes for five viral proteins, named VP1–VP5, with estimated molecular weights of 97 kDa, 41 kDa, 32 kDa, 28 kDa, and 21 kDa, respectively, and is made of two segments [3,4,5]. Segment B (2.8 kb) harbors a single open reading frame (ORF) that encodes the viral RNA-dependent RNA polymerase (VP1), which is involved in viral genome replication and mRNA synthesis and is found both as a genome-linked protein and as a free polypeptide [3,6]. Segment A (3.2 kb) carries two ORFs: the smaller ORF encodes a non-structural 17-kDa protein VP5 responsible for an anti-apoptotic function at the earliest stages of the infection [7]; the larger ORF codes for a 109-kDa precursor polyprotein, which is then cleaved into three separate polypeptides: a capsid protein (VP2), a scaffold protein (VP3) involved in virion morphogenesis, encapsidation, and replication, and a viral protease (VP4) responsible for maturation of VP2 during virus assembly [5,6].

IBDV features a high mutation rate and is also prone to reassortment between the two genome segments, leading to the emergence of numerous genetic variants with profound functional differences [8], including a high antigenic diversity [9]. Two serotypes of IBDV, 1 and 2, are recognized. Only serotype 1 is pathogenic in chickens, while serotype 2 is considered non-virulent [10]. Serotype 1 may be further classified based on pathogenicity and antigenicity: traditionally, strains are divided into classical virulent IBDVs (cvIBDVs), causing typical signs and lesions with low mortality; very virulent IBDVs (vvIBDVs), which trigger clinical forms similar to that induced by cvIBDVs but with higher mortality; and antigenic variants (avIBDVs), which are antigenically divergent from cvIBDVs and vvIBDVs and whose infection is associated to rapid bursal atrophy without inflammatory changes or clinical signs [11].

Although still valuable, this traditional classification is now recognized as inexhaustive, as many atypical IBDV types do not fall into any of the three aforementioned groups. To achieve a more systematic differentiation, several phylogeny-based classification systems have been proposed in recent years. The first classification was proposed by Michel and Jackwood [12] and relies on the sequencing of the hypervariable region of the VP2 (hvVP2), identifying seven different genogroups (G1–G7) within serotype 1. The focus on VP2 is motivated by its well-established role as the principal determinant of antigenicity, as well as its influence on cell tropism and virulence [13,14]. On the other hand, several studies highlighted that VP1 also plays a significant role in pathogenicity determination [15]. Considering both VP2 and VP1 genes thus enables a more comprehensive characterization, and, since they are located in different segments, it also allows to detect reassortment events [16,17]. To this purpose, Islam et al. [18] proposed a classification scheme based on the sequencing of the hvVP2 as well as the B-marker region of the VP1. Nine VP2 (A0–A8) and five VP1 (B1–B5) genogroups have been identified, whose combination allows for a further classification into genotypes (i.e., A1aB1, A1bB1, A2B1, etc.). Further studies established a ninth genotype (A9) [19] and a novel reassortment pattern (A2B3) [20], bringing the total number of recognized genotypes to seventeen.

The application of phylogenetic classification approaches allows the generation of standardized and highly informative data, and thus a deeper understanding even of previously investigated epidemiological contexts. In the case of Algeria, IBD presence is known since the late 1980s [21], but its diagnosis is still mainly based on the clinical signs and gross lesions observed in necropsied birds [22,23]. The few histopathological and morphometric studies of the bursa carried out locally highlighted immunosuppression due to IBD-induced lymphocyte depletion [22,24,25]. Moreover, two serological surveys on IBD were performed during the periods 2014–2016 and 2018–2019 on 30 and 45 Algerian broiler flocks, of which 17.7% and 73.3% were seropositive, respectively [26,27].

As for molecular studies, the first record of VP2 sequencing being used to characterize Algerian strains as vvIBDVs dates back to 2000, as reported by Abed et al. [28]. More recent surveys, also based on just VP2 sequencing, confirmed the circulation of field strains with features consistent with typical vvIBDVs [25,29]. The sole study relying on both VP2 and VP1 sequencing reported the presence not only of vvIBDVs (A3B2), but also of classical attenuated strains (A1B1, presumably of vaccine origin) as well as reassortant strains belonging to genotype A3B1 [28]. Such strains were speculated to be interserotypic reassortants, although the origin of their segment B is currently unknown, but their pathogenicity was found to be comparable to typical vvIBDVs [28].

Despite the value of existing evidence, the scarcity and limited scale of molecular studies performed so far, not complying with the current and more robust classification systems, mean that the epidemiology of Algerian IBDV strains is still poorly understood. Urgent systematic monitoring and surveillance programs are paramount to evaluate the effectiveness of the immunization procedures and to advantageously pinpoint the epidemiological situation of IBD in Algeria. Hence, the objective of this study was to characterize the field IBDV strains circulating in Algerian poultry flocks from different poultry-raising areas in North Algeria, using the current phylogenetic classification scheme.

## 2. Materials and Methods

### 2.1. Sampling

Samples were collected for diagnostic purposes from May 2022 to June 2023 from 70 farms, including 66 broiler and 3 layer flocks, all raised intensively, and a single backyard layer flock. The investigated flocks were sampled between 20 and 56 days of age (mean: 31.5 days), imprinting 4 bursae per flock (total: 280 bursae) on an FTA™ card and processing them as a pool. Farms were located in 41 different communes belonging to 11 districts in Northern Algeria. Twenty flocks were immunized with live intermediate vaccines, 28 with live intermediate plus vaccines, 2 with an immune complex vaccine, and 17 were not vaccinated against IBD. Two of the remaining three flocks were immunized but the applied vaccine was unknown, whereas no information was available for the last one.

### 2.2. Sample Processing and Nucleic Acid Extraction

Portions of FTA card circles imprinted with bursae from the same flock were placed within a single tube with 1.5 mL of 1× PBS. After vortexing for 30 s, 200 μL of each eluate were collected and extraction was performed using the High Pure Nucleic Acids kit (Roche™, Basel, Switzerland) following the manufacturer’s instructions. Sample eluates and extracted nucleic acids were then archived at −80 °C.

### 2.3. Molecular Analysis

Samples were analyzed using two one-step RT-PCR assays, both performed using the SuperScript™ III One-Step RT-PCR System with Platinum™ *Taq* DNA Polymerase kit (Invitrogen™, Waltham, MA, USA). One, conducted with the primer pair 743-1 (5′-GCCCAGAGTCTACACCAT-3′) and 743-2 (5′-CCCGGATTATGTCTTTGA-3′) [30], targeted a portion of the VP2 gene located between positions 737 and 1479 of segment A. The other, which used the primers B-Univ-F (5′-AATGAGGAGTATGAGACCGA-3′) [31] and VP1-shortR (5′-TGGAAACAAAAGCCCGCATG-3′) [32], amplified a portion of the VP1 gene from position 319 to 1070 of segment B. Positive samples were Sanger sequenced using the same PCR primers; then, chromatograms were trimmed in 4peaks (Nucleobytes B.V., Aalsmer, The Netherlands) and used to assemble consensus sequences with ChromasPro (Technelysium Pty Ltd., Helensvale, QLD, Australia).

### 2.4. Phylogenetic Analysis

Two datasets, one for each gene, were built by including the generated sequences along with reference strains. All IBDV sequences of Algerian origin retrievable from GenBank and covering the genomic regions of interest were also downloaded and included in the analysis. Using MEGA X 10.2.4 [33], the datasets were aligned with the MUSCLE method and trimmed to a standard length; then, phylogenetic trees were inferred to characterize IBDV strains based on the classification criteria proposed by Islam et al. [18]. Trees were generated with the Maximum Likelihood Method (1000 bootstraps), adopting the substitution model with the lowest Bayesian information criterion (BIC) score, and visualized with the Interactive Tree Of Life (ITOL) online tool [34].

### 2.5. Statistical Analysis

The efficacy of different vaccination protocols in preventing field strain infections was assessed with the Chi-Square test. The analysis was performed in R 4.4.1 (R Core Team, 2021) [35], setting the significance level at *p* < 0.05. Results were visualized using the vcd package version 1.4-13 [36].

## 3. Results

### 3.1. Phylogenetic Classification

**Figure 1:**
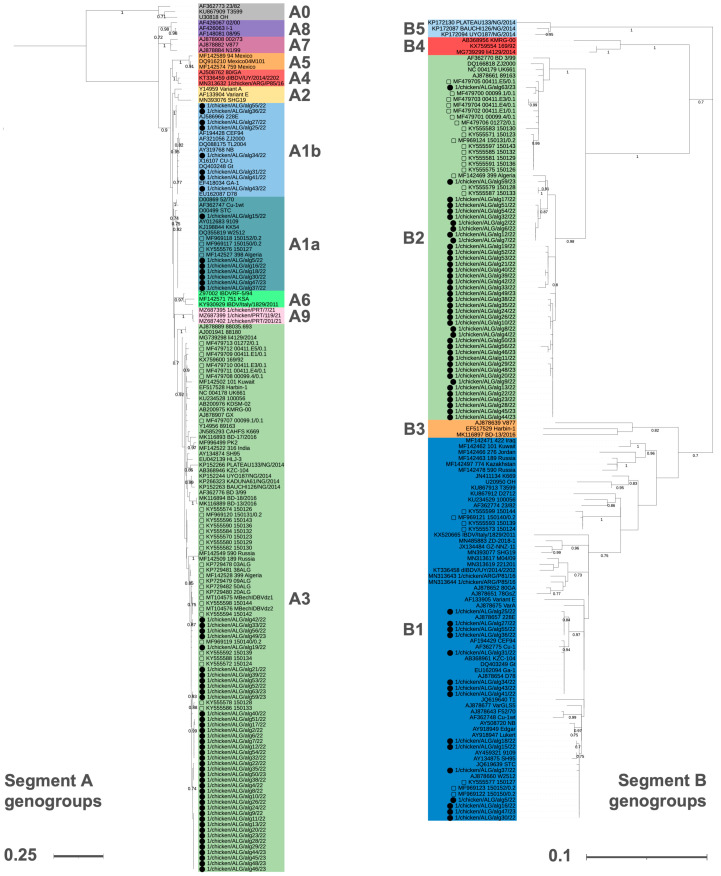
Phylogenetic trees of VP2 (**left**) and VP1 (**right**) sequences, inferred with the Maximum Likelihood Method (1000 bootstraps) adopting the K2+G [37] and GTR+G+I [38] substitution models, respectively. Sequences are color-coded according to their genogroup classification, which refers to the criteria proposed by Islam et al. [18]. Newly obtained sequences are marked with black circles (⬤), whereas other Algerian strains retrieved from GenBank are highlighted with a white square (▢). Node support values are shown only when equal to or higher than 0.7.

Out of the 70 investigated flocks, 55 (78.6%) tested positive to both RT-PCR assays. Based on their VP2 sequences, seven of the detected strains belonged to VP2 genogroup A1a (classical virulent), eight to A1b (classical attenuated), and 40 to A3 (very virulent). All VP1 sequences belonging to A1a and A1b strains clustered within genogroup B1 (classical-like), whereas all A3 viruses belonged to VP1 genogroup B2 (very virulent). Therefore, three genotypes were detected: A1aB1 (seven strains, 12.7% of positive samples), A1bB1 (eight strains, 14.6%), and A3B2 (40 strains, 72.7%) (Figure 1).

### 3.2. Retrieved Genotypes

**Figure 2:**
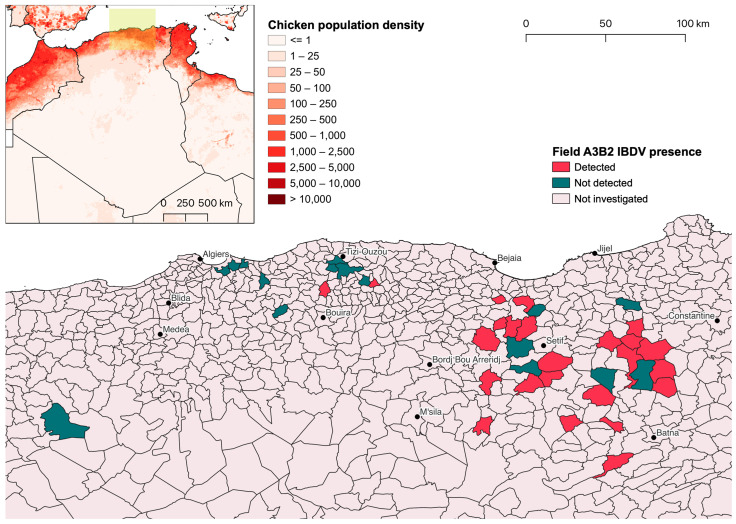
Distribution of field A3B2 strain detections at commune level. The area shown in the main map is highlighted in light green in the inset map in the top left corner, which shows the chicken population distribution according to the Gridded Livestock of the World 4 (GLW4) dataset [39].

Due to their identity with or close relationship to reference vaccine sequences, A1aB1 and A1bB1 strains were considered to be of vaccine origin, whereas A3B2 sequences belonged to field strains and clustered together with previously reported Algerian IBDVs based on both genome segments. In total, A3B2 strains were found in 37 of the 66 sampled broiler flocks, in two out of the three layer farms, and in the only backyard flock, whose geographic distribution is shown in Figure 2. Their sequences were deposited in GenBank under the accession numbers listed in Table 1.

### 3.3. Efficacy of Vaccine Protocol

**Figure 3:**
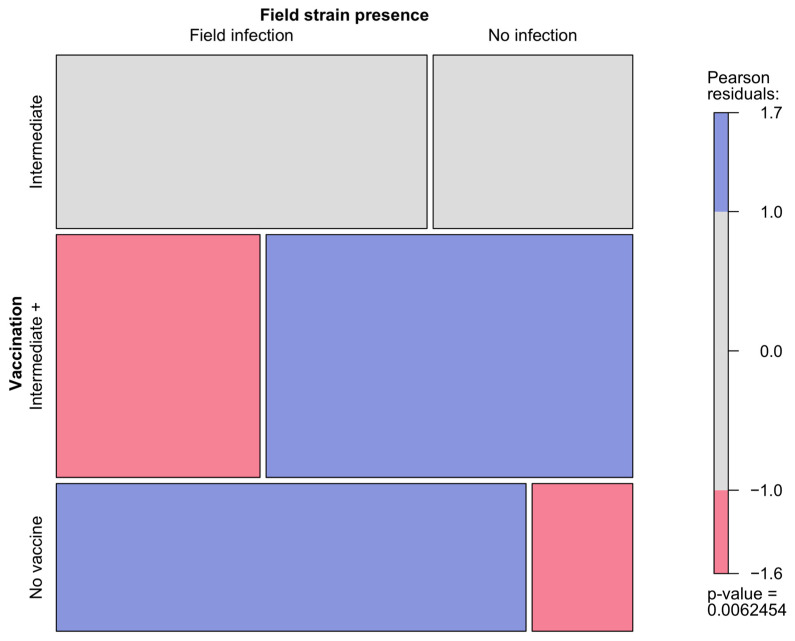
Mosaic plot showing the relationship between the administered vaccination protocol and the detection of field strains. The size of each cell is proportional to the respective count, while the colors represent standardized residuals.

The efficacy in preventing field strain infections was first assessed by comparing vaccinated (25 field strain detections out of 52 samples, 48.1%) and unvaccinated (14 out of 17, 82.4%) flocks, bearing statistically significant differences (X^2^ = 6.1249, *p*-value = 0.013329). Then, a second analysis was conducted considering three vaccination protocols: the use of intermediate vaccines (13 field strain detections out of 20 samples, 65.0%), use of intermediate plus vaccines (10 out of 28, 35.7%), and no vaccination. The use of immune complex vaccines was not considered since they were administered only in two flocks, one of which tested negative whereas the other was positive for the administered vaccine strain. The differences between the three protocols proved significant (X^2^ = 10.1518, *p*-value = 0.006245). In particular, the frequency of field strain detections was higher than expected in unvaccinated flocks, and lower than expected in flocks vaccinated with intermediate plus vaccines (Figure 3).

## 4. Discussion

Despite the endemic status of Gumboro disease in Algeria and the consequent losses [28], the local epidemiological situation has been investigated seldomly, and the IBDV genetic data produced in the country come from a limited number of studies. To address these gaps, an epidemiological survey was conducted based on a novel phylogenetic classification approach, which, by considering portions of both genome segments, allowed a more accurate characterization of IBDV features. For a long time, IBDV molecular investigations, including those performed in Algeria, were almost exclusively based on the VP2 gene, located within segment A, and particularly on its hypervariable region, which is a known marker of virulence, antigenicity, and cell tropism [14,40,41,42,43]. Nonetheless, the role of VP1, encoded by segment B, in the determination of the pathogenicity is also well-established [15,44,45,46]. As a matter of fact, multiple studies have linked the emergence and increased virulence of vvIBDV strains to their segment B, whose origin is unknown [2,47,48,49]. Therefore, VP1 gene analysis helps obtaining complementary genetic information with possible implications for IBDV virulence [50], even in contexts where a wealth of VP2 sequences is available. Moreover, this approach enables the potential detection of reassortment events, which play a relevant role in IBDV evolution [8]. In recent years, this has led to an increased number of reports of naturally occurring reassortants in different parts of the world [20,51,52,53,54], some of which managed to affirm themselves as relevant field types.

Out of the 70 farms sampled in the present study, 55 tested positive (79%). The detected strains belonged to A1aB1 (classical virulent), A1bB1 (classical attenuated), and A3B2 (very virulent). All A1aB1 and A1bB1 IBDVs could be traced back to vaccines used in the country, although they did not necessarily match with the reported vaccination protocol administered in the respective flock. In particular, five of the eight detections of classical attenuated strains (A1aB1) occurred in flocks allegedly immunized with vaccines based on classical virulent IBDVs (A1bB1), and another A1aB1 IBDV was found in an unvaccinated farm. Aside from reporting errors, other possible explanations of these discrepancies might be the spread of vaccine strains among neighboring farms or their persistence from previous cycles following a vaccine change.

All field IBDVs were very virulent strains belonging to genotype A3B2. The first IBD outbreaks in the Arab Maghreb were reported in the late 1980s [21,55] following the emergence of vvIBDVs in Western Africa [56,57] and their further spread to Europe [48,58,59]. The introduction of vvIBDV strains in Algeria might have occurred through wild migratory birds [60,61] or animal trade. The latter hypothesis is supported by the fact that during the 1980s Algerian producers relied on the import of fertilized eggs and day-old chicks, as no breeder farms were present in the country. Since at least 1999, vvIBDVs uninterruptedly represented a threat for the national poultry sector. The field strains characterized in the present study all clustered with previously reported Algerian strains, supporting the continuing circulation of this genotype in the country. Nonetheless, the vvIBDVs detected in a previous survey conducted in Algeria between 2014 and 2015 were also closely related to strains recovered between 2017 and 2019 in Tunisia, and between 2016 and 2017 in Morocco [62,63]. Such findings, easily explained by the geographical proximity and frequency of exchanges between these countries, clearly highlight the transboundary nature of IBDV.

The high prevalence of A3B2 strains suggests an intense viral pressure, likely associated to health consequences and economic losses. Linking field IBD infections to clinical outcomes was outside the scope of the study, as it would have required a more comprehensive diagnostic panel to evaluate the involvement of other primary or secondary pathogens. Nonetheless, compatibly with their very virulent characterization, the detection of field IBDVs was anecdotally associated to bursal lesions, typical IBD signs, and in some cases high mortality rates, although such information was not systematically gathered. Most notably, sample ALG-28 was collected in a layer farm in which mortality was around 85%, possibly as a response to a suspected ND outbreak, whereas ALG-54 came from broilers showing 30% mortality, with no reported clinical manifestations outside of typical IBD lesions. Aside from these two flocks, mortality rates were less severe: samples ALG-51, ALG-52, ALG-53, ALG-55, ALG-56, and ALG-63, for instance, were taken in situations in which the reported mortality was between 2.3 and 7.5%. Above all, what was noted in vvIBDV-infected flocks was the degradation of zootechnical performance. In Algeria, broilers are usually slaughtered between 7 to 8 weeks of age, when the average weight is approximately around 2.8 to 3.2 kg, while in many of the affected farms the average weight was highly heterogeneous and did not exceed 1.9 kg, showing a remarkable growth delay.

Aside from the long-established presence of vvIBDVs, A3B1 strains were also recently reported in Algeria [28]. Showing very virulent-like features at segment A level and an atypical segment B profile close to serotype 2 IBDVs, these viruses were considered interserotypic reassortants, although their pathogenicity closely resembled that of typical vvIBDVs based on experimental infections [28]. This genotype was not detected in the present study, which may suggest that, following the originating reassortment event, the circulation of such strains may have been short-lived, and their presence may not be relevant anymore in Algeria. However, it should be noted that A3B1 IBDVs were detected around the city of Medea, located in Central Algeria [28]. Medea recently became a relevant hub for poultry production, primarily for turkeys, and is also close to the lake of Boughezoul, a place of passage and hibernation for wild migratory birds, possibly explaining the origin of the reassorted segment B. Since this province was not investigated in the present study, another explanation for their absence might be that interserotypic reassortants could still circulate locally without spreading to other parts of the country. This is supported by a recent study conducted in the area of Medea in 2019–2020, which reported the detection of strains having a very virulent-like VP2 showing a high genetic identity (99.3–100%) with the A3B1 IBDVs originally reported in 2015 [25]. Unfortunately, although these results seem to support the persistence of interserotypic reassortants, the unavailability of the respective VP1 sequences does not allow to confirm it with certainty.

Although most samples were collected in broiler farms, field strains were also found in the two layer farms and in the only backyard flock, suggesting that IBDV might affect all sectors of Algerian poultry production. This finding may be explained by the general lack of biosecurity measures and separation between the different types of poultry production in rural zones, with birds of multiple ages and productive types being often raised in the same establishment.

Out of the 67 flocks for which the vaccination status was known, 50 (75%) were actually vaccinated against IBD. The efficacy of vaccination in preventing IBDV infections is supported by the obtained data, as field strain detections were less frequent in immunized flocks. Moreover, statistically significant differences were also found between different vaccine types: not considering immune complex vaccines as they were administered only in two flocks, intermediate plus vaccines were more effective than intermediate ones in preventing field strain infections. Based on this finding, explainable with the higher maternally derived antibodies (MDAs) breakthrough power of intermediate plus vaccines, less attenuated vaccines might be preferred in epidemiological contexts like Algeria where infectious pressure is high.

Although crucial to curb infectious pressure, it should be noted that preventing field strain infections is not the primary aim of IBD vaccination, whose outcome should also be assessed based on the prevention of clinical signs and immunosuppression. Nonetheless, vaccination efficacy appeared suboptimal regardless of the vaccine type. Considering that in Algeria live vaccination is mostly carried out at around 14 days of age without first assessing the level of MDAs, immunization could likely be improved through careful vaccination timing. Moreover, curating all aspects of drinking water administration (i.e., vaccine reconstitution, quality of the water, water intake) could also lead to better vaccination results [64].

## 5. Conclusions

Very virulent A3B2 strains appear to extensively and persistently circulate in Algeria, confirming their role as the predominant field threat in the country, whereas other field IBDV types, such as the recently reported A3B1 reassortants, were not detected. Based on the collected evidence, current vaccination efforts appear to partially reduce IBDV infections compared to unvaccinated flocks, but further attention appears needed in light of the high infectious pressure. The obtained findings suggest that all flocks should be properly immunized. Failing to vaccinate does not represent a liability just for the unvaccinated farm, but for the entire epidemiological context, as it will facilitate field IBDV persistence and spread to other facilities. Although intermediate plus vaccines appeared preferable, likely due to their higher breakthrough power, other factors, such as careful administration and accurate immunization timing, coupled with rigorous biosecurity, are also important for effective control. Finally, the present results clearly demonstrate the importance of conducting attentive and continuous monitoring activities according to standardized classification systems, which allow to confidently assess the epidemiological situation and react accordingly.

## Figures and Tables

**Table 1:** List of detected A3B2 field strains along with anamnestic details and the respective accession numbers.

Sample ID	Productive Category	Vaccination Protocol	VP2 Accession No.	VP1 Accession No.
ALG-2	Broilers	No vaccination	PQ439956	PQ439996
ALG-4	Broilers	No vaccination	PQ439957	PQ439997
ALG-6	Broilers	No vaccination	PQ439958	PQ439998
ALG-7	Broilers	Live intermediate	PQ439959	PQ439999
ALG-8	Broilers	No vaccination	PQ439960	PQ440000
ALG-9	Broilers	No vaccination	PQ439961	PQ440001
ALG-10	Broilers	No vaccination	PQ439962	PQ440002
ALG-11	Broilers	No vaccination	PQ439963	PQ440003
ALG-12	Broilers	No vaccination	PQ439964	PQ440004
ALG-13	Broilers	No vaccination	PQ439965	PQ440005
ALG-17	Broilers	Live intermediate plus	PQ439966	PQ440006
ALG-19	Broilers	Live intermediate	PQ439967	PQ440007
ALG-20	Broilers	Live intermediate	PQ439968	PQ440008
ALG-21	Broilers	Live intermediate	PQ439969	PQ440009
ALG-22	Broilers	No vaccination	PQ439970	PQ440010
ALG-23	Backyard layers	Unknown	PQ439971	PQ440011
ALG-24	Broilers	Live intermediate	PQ439972	PQ440012
ALG-26	Broilers	Live intermediate plus	PQ439973	PQ440013
ALG-28	Layers hens	Live intermediate plus	PQ439974	PQ440014
ALG-29	Broilers	Live intermediate plus	PQ439975	PQ440015
ALG-32	Broilers	Live intermediate	PQ439976	PQ440016
ALG-33	Broilers	Live intermediate	PQ439977	PQ440017
ALG-35	Broilers	Unknown	PQ439978	PQ440018
ALG-38	Broilers	Live intermediate	PQ439979	PQ440019
ALG-39	Broilers	Unknown	PQ439980	PQ440020
ALG-40	Broilers	Live intermediate	PQ439981	PQ440021
ALG-42	Broilers	Live intermediate plus	PQ439982	PQ440022
ALG-44	Broilers	No vaccination	PQ439983	PQ440023
ALG-45	Broilers	No vaccination	PQ439984	PQ440024
ALG-46	Broilers	No vaccination	PQ439985	PQ440025
ALG-48	Broilers	Live intermediate	PQ439986	PQ440026
ALG-49	Broilers	Live intermediate plus	PQ439987	PQ440027
ALG-50	Broilers	No vaccination	PQ439988	PQ440028
ALG-51	Layers hens	Live intermediate	PQ439989	PQ440029
ALG-52	Broilers	Live intermediate	PQ439990	PQ440030
ALG-53	Broilers	Live intermediate	PQ439991	PQ440031
ALG-54	Broilers	Live intermediate plus	PQ439992	PQ440032
ALG-56	Broilers	Live intermediate plus	PQ439993	PQ440033
ALG-59	Broilers	Live intermediate plus	PQ439994	PQ440034
ALG-63	Broilers	Live intermediate plus	PQ439995	PQ440035

## Data Availability

The original contributions presented in the study are included in the article; further inquiries can be directed to the corresponding author.
